# Investigation of Current Effect of Suspended Graphene Pressure Sensor

**DOI:** 10.3390/ma18122801

**Published:** 2025-06-14

**Authors:** Haowei Mi, Run Qi, Pengcheng Li, Ningning Su, Junqiang Wang

**Affiliations:** 1 Academy for Advanced Interdisciplinary Research, North University of China, Taiyuan 030051, China; sz202206183@st.nuc.edu.cn (H.M.); s202206031@st.nuc.edu.cn (R.Q.); sz202206094@st.nuc.edu.cn (P.L.); 2 Shanxi Key Laboratory of Graphene Sensing Materials and Devices, North University of China, Taiyuan 030051, China; 3 School of Instrument and Electronics, North University of China, Taiyuan 030051, China; 4 School of Semiconductors and Physics, North University of China, Taiyuan 030051, China

**Keywords:** suspended graphene, current effect, piezoresistive effect, pressure sensor

## Abstract

The current effect of passive devices is crucial for device testing. The current effect of a suspended graphene pressure sensor in the range of 0–2 mA is studied in this paper. The results show that the resistance of graphene films and the piezoresistive effect of devices exhibit stable performance within the current threshold range of 400 μA and 300 μA, respectively. Auger electron spectroscopy and Raman spectroscopy tests indicate that the resistance of graphene increases first and then decreases at high current intensity, resulting from the electrostatic adsorption of oxygen atoms in the initial phase of electrification and the Joule-induced desorption in the later phase. This study presents guiding significance for the electrical testing of suspended graphene devices.

## 1. Introduction

Graphene, composed of carbon atoms arranged in a hexagonal lattice, is an ultra-thin two-dimensional material. Due to its exceptional properties, graphene has garnered widespread attention in the international research community. These properties include an extremely high Young’s modulus of up to 1 TPa [1,2], a fracture strength of 130 GPa [3], and an electron mobility of up to 2.5 × 10^5^ cm^2^/V·s [4,5]. The unique structure and properties of graphene make it an ideal material for microelectromechanical systems [6,7], particularly for the fabrication of highly sensitive piezoresistive materials [8,9,10]. In 2015, a report introduced the development of an ultra-high-sensitivity and compact graphene-based diaphragm pressure sensor [11]. In 2016, a highly sensitive capacitive pressure sensor utilizing an ultra-large suspended graphene membrane was successfully realized [12]. By 2020, the fabrication of sensitive capacitive pressure sensors based on arrays of suspended graphene membranes had been achieved, exhibiting a remarkable sensitivity of up to 47.8 aF Pa^−1^ mm^−2^ [13]. In 2021, a Pirani pressure sensor based on suspended graphene membranes with a small footprint was realized [14]. Graphene pressure sensor devices, as passive components, generate thermal effects when electrified [15,16,17]. When current passes through graphene, electron–lattice interactions convert energy into heat [18]. Such thermal effects can cause the temperature of the graphene to rise, thereby affecting its electrical properties, e.g., reducing electron mobility, which in turn affects the response speed and sensitivity of the sensor [19]. Under certain current conditions, the thermal effect can even lead to the desorption of adsorbates on the graphene surface [20]. Moreover, localized overheating can lead to structural damage in the graphene, decreasing its mechanical strength and electrical conductivity. Electromigration refers to the phenomenon in which atoms or impurities within the graphene migrate under the influence of an electric field at high current densities. This migration increases the number of structural defects in the graphene, further affecting its electrical performance by increasing resistivity and potentially causing the graphene to fracture or delaminate, severely impacting the reliability and lifespan of the sensor [21,22]. During the electrification process, structural damage to the graphene may occur due to the thermal effects of the current and electromigration. For instance, localized overheating can cause the lattice structure of the graphene to twist or fracture, while atom migration caused by electromigration can disrupt the integrity of the graphene, making it more susceptible to delamination or layer separation. These structural damages not only affect the electrical performance of the graphene but may also limit its application in sensors [23]. Moreover, the thermal effect produced when the graphene is electrified can cause it to expand thermally. Thermal expansion alters the lattice parameters of the graphene, affecting its electronic structure and electrical properties [24,25,26]. Recent studies on suspended 2D materials have revealed unique thermal transport properties critical for device applications. For instance, Seol et al. demonstrated that suspended graphene exhibits exceptionally high thermal conductivity (>2000 W/m·K) due to suppressed phonon scattering [27], while an analogous work on TMDs identified boundary scattering as a dominant factor in current-dependent thermal resistance [28]. Notably, hBN sheets show remarkable thermal stability even under extreme current densities [29], suggesting material-specific responses to Joule heating. However, despite these established thermal–electrical couplings in 2D materials, current studies on graphene pressure sensors have largely overlooked the systematic investigation of current-dependent behaviors in suspended graphene architectures—a critical knowledge gap.

The thermal effects generated when graphene is electrified can significantly affect its interaction with the substrate, thereby influencing the performance of the graphene [30,31]. Firstly, the high thermal conductivity of graphene allows it to quickly conduct heat when electrified, but when graphene is in contact with a substrate, its thermal conductivity is significantly affected [32]. Secondly, the interaction between graphene and the substrate can also affect its electronic structure and electrical performance [33,34]. The thermal expansion coefficient of the substrate differs from that of graphene, and the thermal effects produced during electrification can lead to changes in the interfacial stress between the substrate and graphene. Such stress changes may alter the electron mobility and resistivity of graphene [35].

**Figure 1:**
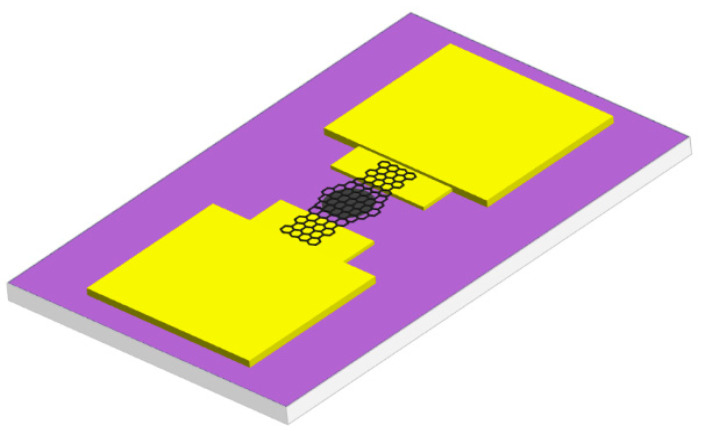
Diagram of a suspended graphene pressure sensor.

In this study, the effect of current on the resistance and piezoresistive properties of the suspended graphene device is studied. The preview of the device structure is shown in Figure 1.

The suspended graphene structure, being free from substrate interactions, provides an ideal platform for investigating the intrinsic properties of the material. By applying different current densities to these devices, we aim to elucidate how current affects the resistance and piezoresistive response of graphene. This research is expected to contribute to the development of more efficient and reliable graphene-based electronic devices.

## 2. Sensor Fabrication

To mitigate the impact of the thermal effects generated by electrified graphene on the substrate, this study has designed a suspended graphene pressure device with a bottom cavity linked to an upper opening. This structural design aims to minimize direct contact between the graphene and the substrate, thereby reducing the substrate’s interference with the thermal conduction pathways of the graphene. By employing this approach, the performance changes in the graphene itself under the thermal effects of electric current can be more accurately characterized and demonstrated. Specifically, the suspended structure reduces the thermal coupling between the graphene and the substrate, allowing the thermal conduction characteristics of the graphene to more closely approximate its ideal performance in a freely suspended state. Moreover, this design effectively diminishes the substrate’s influence on the electronic structure and electrical properties of the graphene.

**Figure 2:**
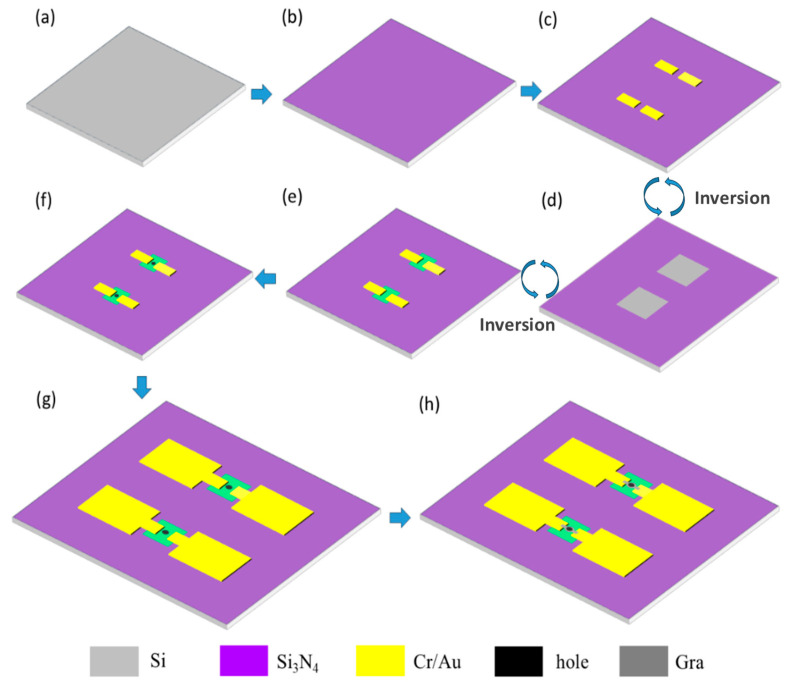
Structure and fabrication process of the sensors. (**a**) The silicon substrate is cleaned. (**b**) LPCVD deposition of silicon nitride. (**c**) Sputtering growth of Cr/Au. (**d**) RIE etching of silicon nitride on the back side of the silicon wafer. (**e**) Wet etching in KOH solution. (**f**) RIE etching to open holes in the silicon nitride. (**g**) Electrode elevation. (**h**) Transfer of graphene and patterning.

**Figure 3:**
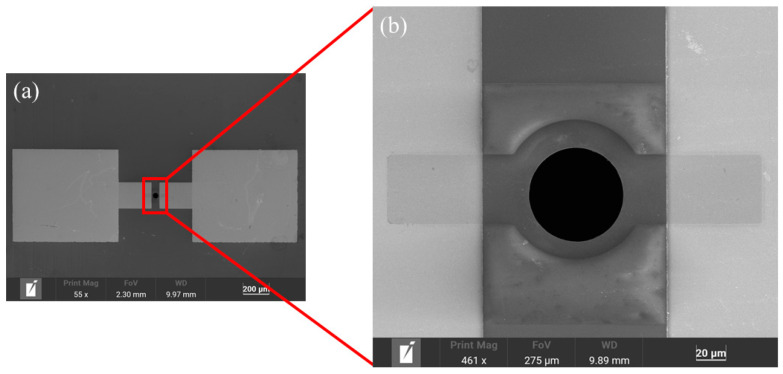
SEM images of the sensors. (**a**) Suspended graphene sensor chip. (**b**) Magnified image of the graphene unit.

The fabrication process of the device is shown in Figure 2. Fabrication begins with the deposition of a 200 nm silicon nitride insulating layer on both sides of the silicon wafer (200 μm, N-type, 100 crystal orientation) via low-pressure chemical vapor deposition (LPCVD) at 785 °C for 2 h. Then, a 15 nm/25 nm Cr/Au electrode layer was sputtered on the front side of the silicon wafer. The base pattern was fabricated using NR9-3000PY negative photoresist (Futurrex, Inc, Boonton, NJ, USA), with spin-coating parameters of 600 rpm for 6 s and 3000 rpm for 40 s, then baked at 150 °C for 120 s. The photolithography process included MA6-1 mask alignment exposure for 9 s, post-exposure baking at 100 °C for 90 s, and development in TMAH 2.38% solution for 15 s, followed by O_2_ plasma treatment (150 sccm, 200 W, 2 min) and final lift-off processing with acetone. Thirdly, using AZ5214 positive photoresist (Clariant, AG, Muttenz, Switzerland), a cavity with a depth of 200 nm and a width of 372 μm was fabricated on the back of the silicon wafer through a combination of reactive ion etching (RIE) and lithography for 4 min. The resist was spin-coated at 600 rpm for 6 s, followed by 4000 rpm for 30 s, then baked at 95 °C for 90 s. The lithography process involved MA6-1 mask alignment exposure for 7 s and development in 2.38% TMAH solution for 40 s. Fourth, the silicon wafer was immersed in an 85 °C KOH:H_2_O (1:1) solution for a 2 h wet etching process, selectively removing the exposed silicon until the etching reached the bottom surface of the front-side silicon nitride layer (indicated by the green coloration in Figure 2). Fifth, a through hole with a diameter of 20 microns and a depth of 200 nm was formed at the center of the silicon wafer electrode via reactive ion etching for 4 min to establish a connection with the lower cavity. The photolithography process was the same as that employed in the second step. Finally, monolayer graphene was transferred to the chip surface through wet transfer and patterned using oxygen plasma microwave RIE and AZ5214 positive gel to form a circular film with a diameter of 40 μm at the center; the lithography process remained unchanged, and two 20 μm wide graphene conductive bands extended to the Cr/Au electrode. The morphology of the fabricated device was characterized by scanning electron microscopy (SEM) under optimized imaging conditions (accelerating voltage: 5 keV; working distance: 9.89 mm), with representative images shown in Figure 3 (print magnification: 461×; field of view: 275 μm) clearly showing the circular graphene diaphragm suspended over the 20 μm cavity.

In particular, Figure 3b clearly reveals the transferred graphene surface, demonstrating a clean and intact structure with no observable PMMA residue. These results confirm that our transfer process effectively avoids polymer contamination, providing a reliable foundation for subsequent device performance studies.

## 3. Experiment

### 3.1. Voltammetry Characteristics of Graphene Devices

**Figure 4:**
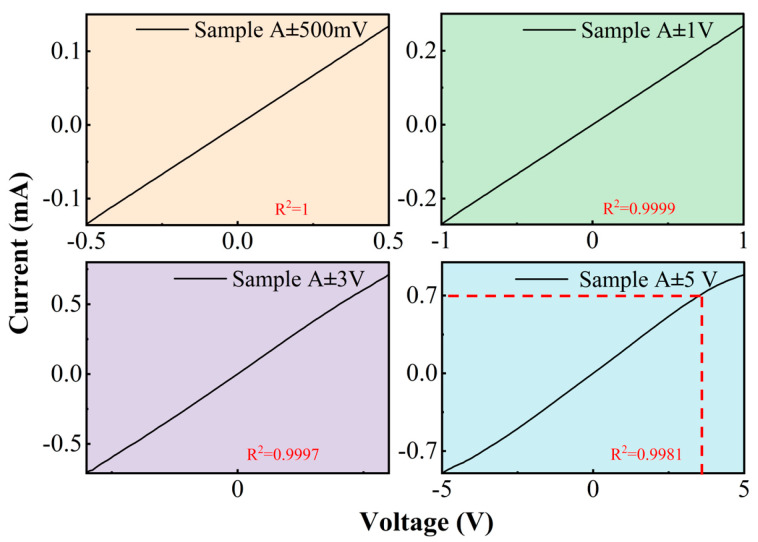
Current–voltage (I–V) characteristic curves of the device.

All experimental measurements were conducted under controlled ambient conditions (atmospheric pressure: 1 atm; temperature: 23 ± 0.5 °C; relative humidity: 45 ± 5%), and the graphene was purchased from XFNANO, Nanjing, China (product no. 7440-44-0, ACS Material, LLC, Pasadena, CA, USA). To systematically evaluate the performance stability of graphene devices, we conducted current–voltage (I–V) characteristic measurements under ambient atmospheric pressure using a PW-600 (Advanced Probing Systems, Inc, Santa Clara, CA, USA) integrated device analyzer. The system automatically acquired 100 data points across the specified voltage range, obtaining precise resistance characteristic curves through instantaneous measurements. The results show that the device displays good I–V characteristics at ±500 mV, ±1 V and ±3 V, and the correlation coefficient R^2^ remains almost unchanged, indicating that the device exhibits high stability at low current intensity. However, across multiple tested devices (n = 8), we consistently observed that when the ±5 V current size is 700 μA, the slope of the device on the I–V curve shows a clear and sudden drop, and the correlation coefficient R^2^ = 0.9981, which may be due to enhanced electron–phonon scattering caused by transient Joule heating in graphene, as shown in Figure 4.

### 3.2. Current Resistance Characteristics

Three groups of different devices (n = 15, R = 4.1 kΩ ± 300 Ω) were employed, using constant current sources of 100 μA, 400 μA, 700 μA, 1 mA, and 2 mA for characterization.

**Figure 5:**
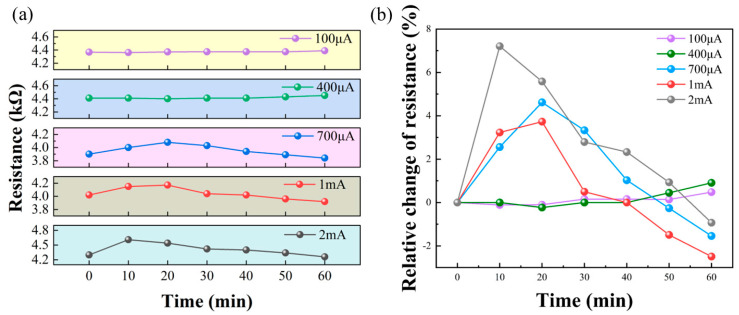
(**a**) Change in device resistance with time at different current intensities. (**b**) The variation in the rate of change in resistance with time.

The resistance data is recorded every 10 min for 1 h to calculate the relative change. As shown in Figure 5a,b, at 100 μA and 400 μA, the resistance remains basically stable for 1 h, with a maximum change of less than 1%.

In contrast, at 700 μA, 1 mA, and 2 mA conditions, the resistance of graphene showed a trend of first increasing and then continuing to decrease. It is worth noting that at the 2 mA condition, the resistance anomaly occurs earlier.

**Figure 6:**
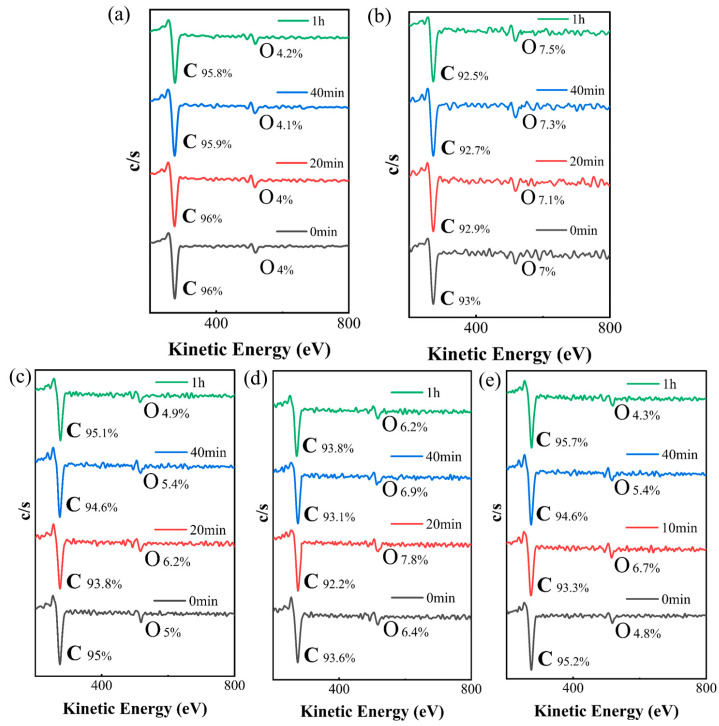
Auger electron spectra of graphene at different timepoints: (**a**) 100 μA; (**b**) 400 μA; (**c**) 700 μA; (**d**) 1 mA; (**e**) 2 mA.

In order to further investigate the resistance characteristics of graphene devices under different current conditions, we performed vacuum Auger electron energy spectroscopy (AES) analysis for each device, mainly characterizing the changes in the carbon (C) and oxygen (O) elements. AES analysis results under different current intensities are shown in Figure 6.

**Figure 7:**
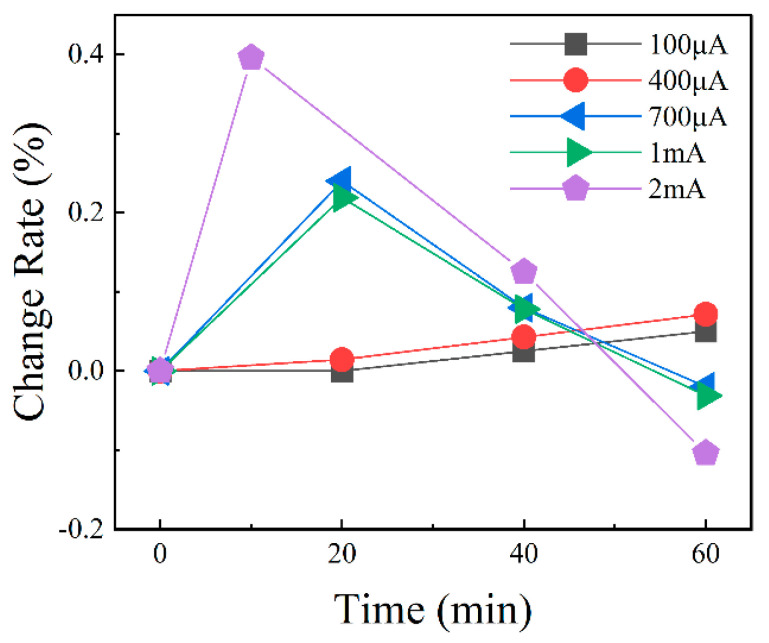
The change in oxygen atom rate in graphene under different currents.

In devices subjected to 100 μA and 400 μA currents, the oxygen content exhibited a slight but insignificant increase. This phenomenon occurred because the Joule heating generated at these low current intensities was insufficient to induce impurity desorption, while the persistent current-induced adsorption resulted in a marginal elevation of oxygen content. However, the oxygen content of the devices under the 700 μA, 1 mA, and 2 mA currents increases noticeably during the resistance increase stage. This phenomenon is consistent with the increase observed in the devices at the 100 μA and 400 μA currents, and may be related to the electrostatic adsorption effect. In this effect, the external electric field enhances the adsorption capacity of the surface of the device for oxygen atoms in the air, resulting in an increase in the O content of graphene. The adsorbed oxygen molecules or atoms will introduce additional scattering barriers, increasing the scattering probability of the carriers (electrons or holes), thereby reducing the mobility and causing the resistance to rise. During the resistance decline stage, the oxygen content gradually decreases and eventually drops below the initial level, as shown in Figure 7.

This result may be due to the fact that as the electrification time is prolonged, the state of continuous high temperature inside the graphene provides enough energy for the adsorbed and inherent oxygen atoms to separate from the surface, resulting in desorption. This process is consistent with a downward trend in resistance, as the downward trend of resistance in the 2 mA device emerged earlier, further supporting the hypothesis that the desorption of the adsorbed material leads to a decrease in resistance.

**Figure 8:**
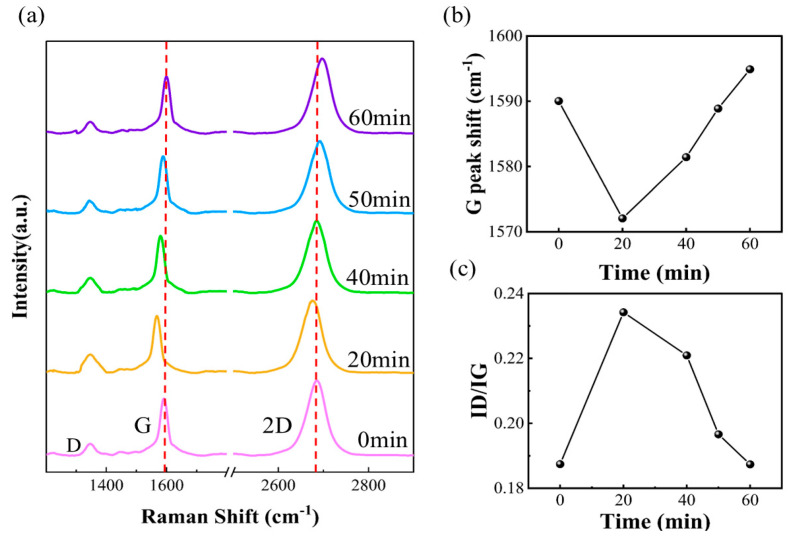
(**a**) Raman spectra of graphene at 1 mA. (**b**) The curve of ID/IG ratio over time. (**c**) The curve of G peak wave number over time.

Through AES analysis, we observed that the variation in oxygen content was significantly correlated with the resistance characteristics. To further investigate the effect of long-term electrification under a high current of 1 mA on the adsorption and desorption behavior of impurities in graphene, Raman spectroscopy was performed using a 532 nm laser with a surface power of 2 mW and a 200 μm aperture, as shown in Figure 8a. The results are presented in Figure 8a.

The experimental results show that the intensity of peak D increases significantly, and peak G shifts to a lower frequency (redshift) 20 min after the power is switched on. After calculation, the maximum temperature can reach 132 °C. This phenomenon indicates that the defect density of graphene increases, possibly due to the electrostatic adsorption effect that causes O and other impurity elements to accumulate on the graphene surface, introducing additional scattering centers and thus enhancing the Raman signal associated with the defect. However, after 20 min of power-on, the intensity of peak D gradually weakened, and peak G shifted to a higher frequency (blueshift), as shown in Figure 8b. Meanwhile, the initial redshift of the 2D peak reflects the strain generated by the temperature increase in graphene and oxygen adsorption, while the subsequent blueshift is due to strain release during high-temperature desorption and thermal shrinkage. In addition, the ratio of D-peak intensity to G-peak intensity (ID/IG) in the Raman spectrum of graphene is an important parameter for characterizing the defect density and structural properties of graphene. As shown in Figure 8c, the ID/IG ratio gradually increased before 20 min of energizing and decreased significantly after 20 min of energizing. This change clearly reflects the dynamic evolution of the graphene lattice structure and the change in defect density. The evolution of these Raman spectral features is highly consistent with the O content changes observed in AES analysis, indicating that electrostatic adsorption causes the accumulation of impurity elements on the graphene surface during the initial phase of electrification, increasing the defect density. At the later stage of electrification, as the high temperature inside graphene persists, the adsorbed oxygen atoms gain enough energy to separate from the surface, leading to desorption and a reduction in defect density. This process strongly supports the resistance change mechanism of electrostatic adsorption in the initial stage and desorption in the later stage under the condition of high current intensity.

### 3.3. Current Piezoresistive Characteristics

**Figure 9:**
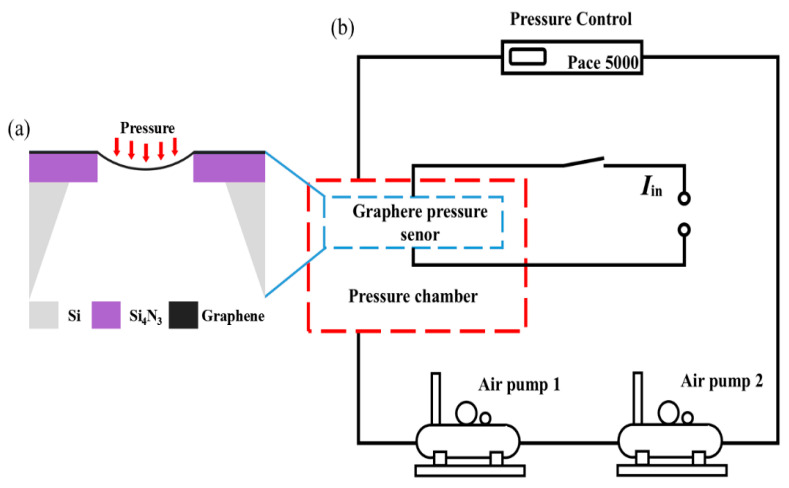
(**a**) Schematic diagram of the test principle. (**b**) Schematic diagram of the test equipment.

In order to study the piezoresistive effects of graphene devices at different currents, whether or not the piezoresistive effects of the devices themselves are stable must first be verified. In the experiment, the device is first placed in a closed chamber, and the pressure difference in the chamber is controlled by the intake and exhaust of the dual pump system so that the air in the sealed chamber is compressed or expanded to provide the chamber pressure. The pressure is monitored by the pressure sensor (Model ALKC600, provided by Alek Measurement & Control, Shanghai, China) in real time. The Druck PACE 5000 pressure controller (Baker Hughes, Leicester, UK) is used to precisely regulate chamber pressure. The test principle and equipment are shown in Figure 9a,b.

The initial pressure in the sealed chamber was set to 101 kPa, and the piezoresistive response of the sensor was systematically characterized under applied pressure differences (0–60 kPa). For the resistance measurements, we employed a digital multimeter (Keysight 34465A, Keysight Technologies, Santa Rosa, CA, USA) with a 0.1 V test voltage instead of a constant current source, limiting the current through the graphene sensing element to approximately 20 μA—a negligible level that can be effectively considered as zero current for all practical purposes in this experiment, thereby minimizing the Joule heating effects while maintaining measurement integrity.

**Figure 10:**
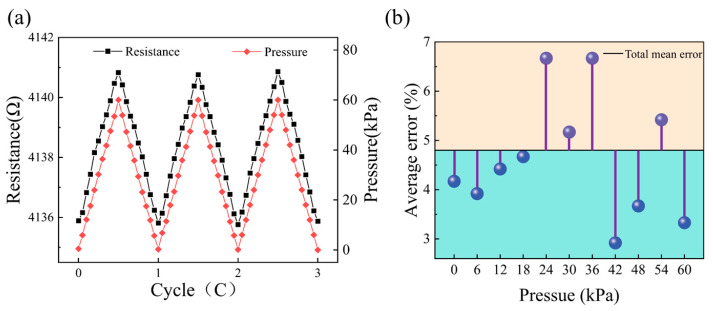
(**a**) Resistance changes in graphene over three cycles. (**b**) Average resistance error over three cycles.

The cyclic test data for the piezoresistive effect of the graphene devices are shown in Figure 10a.

Under 60 kPa pressure, the maximum resistance change rate (about 0.13%) is observed, and the pressure sensitivity is 2.028 × 10^5^ kPa^−1^ which can be obtained by

(1) $$GF=\frac{\left(\frac{\Delta R}{R_{0}}\right)}{\epsilon }$$

Pressure converts to strain via plate theory, as shown below:

(2) $$P=\frac{4\epsilon Eh^{2}}{3r^{2}\left(1-\nu^{2}\right)}$$

where *E* = 1 TPa, *h* = 0.34 nm, *r* = 20 μm, and *ν* = 0.17.

Combining (1) and (2) establishes a closed-loop prediction model:

(3) $$P=\frac{4Eh^{2}}{3r^{2}\left(1-\nu^{2}\right)\cdot GF}\cdot \left(\frac{R-R_{0}}{R_{0}}\right)$$

**Figure 11:**
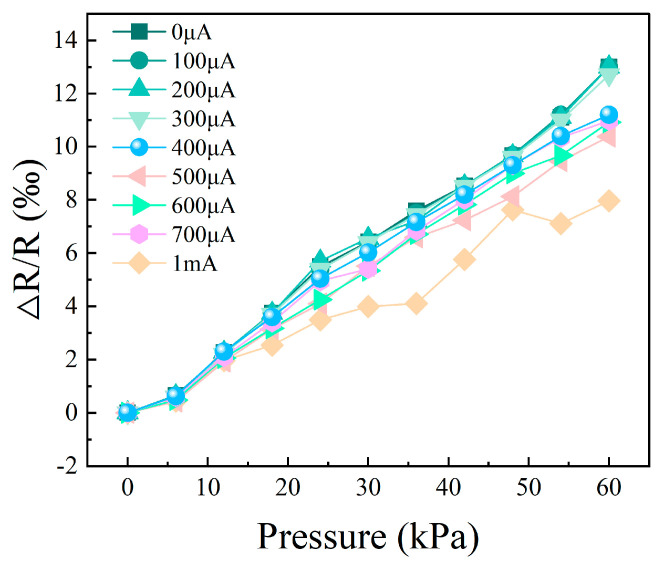
Curve of change rate in current resistance at different intensities.

In order to represent the stability of the device, the average value of the data of each pressure node is taken as the true value, and the average error of each node is calculated, as shown in Figure 10b. The results show that the total average error of the device is 4.8%. A result of less than 5% confirms the stability of the device. In order to avoid the direct effect of high intensity current on the graphene resistance, we selected a current range of less than 700 μA for piezoresistive comparison experiments, according to the current–voltage characteristics shown in Figure 4. As shown in Figure 11, the resistance change rate of graphene remains relatively stable in the current range of 0 μA to 300 μA. When the current reaches 400 μA and above, the rate of resistance change decreases with increasing current, and this trend is more obvious at higher pressures. This phenomenon may be related to the Joule thermal effect caused by the increase in current: the enhanced thermal effect may change the lattice structure of graphene and its response to mechanical stress, thus weakening the piezoresistive effect. The experiments show that the piezoresistive properties of graphene remain relatively stable at a current of less than 300 μA. However, when the current exceeds 300 μA, the change rate and stability of the piezoresistive effect resistance gradually decrease. These results further support the conclusion that the current-induced thermal effect significantly affects the piezoresistive properties of graphene at high current intensity.

## 4. Conclusions

In summary, we fabricated a suspended graphene pressure sensor and systematically studied its current-dependent behavior within the range of 0–2 mA. The resistance behavior of this device remains stable below 400 μA and shows a trend of first increasing and then decreasing above 700 μA. The analysis through AES and Raman spectroscopy indicates that this phenomenon is attributed to the dynamic oxygen adsorption–desorption interaction occurring on the surface of suspended graphene. At the initial stage of electrification, electrostatic adsorption leads to the accumulation of oxygen atoms, increasing the defect density and thereby increasing the resistance. In the later stage, the sustained high-temperature state triggers thermally activated oxygen desorption—reducing defect scattering resistance, while simultaneously generating vacancy clusters that may establish alternative conductive pathways, collectively contributing to the observed resistance decrease. These results highlight the crucial role of impurity kinetics in regulating graphene’s electrical properties. Of course, although the temperature used in this study is lower than the typical annealing threshold, long-term current stress can improve the graphene–metal contact and reduce the contact resistance through thermal annealing. Therefore, the decrease in resistance noted in this paper does not negate the fact that there are some factors remaining for the improvement of the graphene–metal contact. However, our 60 min operational tests (aligned with industry-standard burn-in protocols) demonstrate applicability in both chemical plant pressure monitors, requiring <1% drift over 8 h shifts and continuous intracranial pressure monitoring, demanding 12+ h of stable operation. In addition, under the same pressure conditions, due to the thermal effect of the current, the resistance change rate of the suspended graphene device fluctuates with the increase in the current, and its stability decreases. It is worth noting that a current of 300 μA or less has almost no effect on the piezoresistive characteristics, making this range an ideal choice for reliable device operation. This research provides insights for optimizing the electrical test scheme of suspended graphene devices and emphasizes the importance of precise current control in enhancing the reliability of the devices.

## Data Availability

The original contributions presented in this study are included in the article. Further inquiries can be directed to the corresponding author(s).

## Abbreviations

The following abbreviations are used in this manuscript:

LPCVD	Low Pressure Chemical Vapor Deposition
RIE	Reactive Ion Etching
SEM	Scanning Electron Microscope
PMMA	Polymethyl Methacrylate
AES	Auger Electron Spectroscopy
ID/IG	Intensity Ratio of D-band to G-band
